# Coordinated Synthesis of Pigments Differing in Side Chain Length in *Monascus purpureus* and Investigation of Pigments and Citrinin Relation

**DOI:** 10.1021/acs.jafc.4c09653

**Published:** 2025-01-10

**Authors:** Marketa Husakova, Barbora Branska, Petra Patakova

**Affiliations:** Department of Biotechnology, University of Chemistry and Technology Prague, Technicka 5, Prague CZ166 28, Czechia

**Keywords:** *Monascus purpureus*, pigments, citrinin, stress conditions, regulation of secondary metabolites biosynthesis

## Abstract

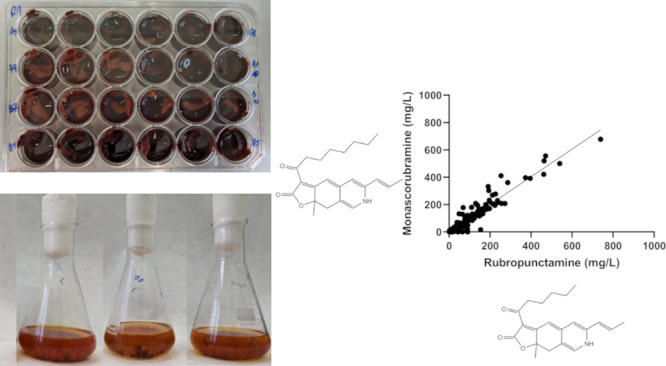

The *Monascus* fungi have traditionally been used in Asia for food coloring. Unfortunately, the most well-known species, *Monascus purpureus*, very often produce mycotoxin citrinin in addition to pigments, which poses a significant problem for the use of pigments in foods. There is a step in pigment biosynthesis where a side chain of five or seven carbons is attached to the tetraketide, the product of polyketide synthase, resulting in the formation of pigments in pairs. Further, it is still unclear whether pigment and citrinin biosyntheses are related or independent. Therefore, this study is focused on the relationship between pigment and citrinin production and pigment analogues that differ in side chain length, all evaluated by the Spearman correlation test. To generate sufficient data, *Monascus purpureus* DBM 4360 was cultivated with different carbon and nitrogen sources and under osmotic stress induced by glucose and/or sodium chloride. The study reveals a very strong correlation between the production of five- and seven-carbon side chain pigments under all culture conditions tested for all three groups, yellow, orange, and red pigments. The correlation between pigments and citrinin depended on the group assessed and ranged from fair to very strong. While the coordinated synthesis of pigment analogues in pairs has been clearly confirmed, the relationship between pigment and citrinin production was unfortunately neither confirmed nor refuted and must be the subject of further research.

## Introduction

Fungi of the so-called Rubri section of *Monascus*,^1^ which are characterized by the attractive red, pink, or orange coloration of their mycelia and the neighborhood of their colonies, have a long history of safe use in the food industry in various oriental countries, such as China, Japan, Korea, or Thailand.^2^ The best known species is *Monascus purpureus*, which produces oligoketides, especially red pigments, used in different food products, as part of a microbial culture for the production of special types of rice vinegar, wine, or brandy, or for the production of red fermented rice containing monacolin K.^2,3^ Natural red pigments produced by *Monascus* fungi would certainly be an attractive alternative to both artificial red food dyes and nitrite salt used in the meat industry.^4^ However, this potential is limited by the fact that *Monascus* fungi frequently, though not always, also produce the mycotoxin citrinin along with the pigments. Citrinin is recognized as a serious contaminant, produced by different fungi including particular *Monascus* strains.^5^

Pigments and citrinin, secondary metabolites of *Monascus*, are oligoketides and are frequently formed under the same conditions as in a mixture. Nevertheless, it seems that they can be produced independently, because isolates or mutants that form pigments without citrinin^6,7^ or citrinin without pigments^8^ have been described. In addition, different biosynthetic pathways were proposed for them.^9^ Pigment biosynthesis is based on polyketide synthase, MrpigA, producing the tetraketide intermediate which is further elaborated by incorporation of 3-oxo-octanoic or 3-oxo-decanoic acid, generated by fatty acid synthase(s) and by the action of tailoring enzymes, such as MrpigC, MrpigN, MrpigD, MrpigE, and others.^10^ Not all steps in biosynthesis are confirmed, and the design of the pathway undergoes continuous improvements and refinements.^11,12^ In the biosynthesis of citrinin, a citrinin-specific polyketide synthase, PksCT, was identified in *Monascus*, which also produces a tetraketide intermediate, and the completion of citrinin biosynthesis also requires a couple of tailoring enzymes.^12^ The question arises whether the two biosynthetic pathways, for pigments and citrinin, are integrated or jointly regulated or whether they are mutually independent. The question has recently been discussed in a review^13^ but with an unclear conclusion.

Various approaches can be applied, including media optimization^14,15^ or exposure to different stress factors^16^ in order to affect the production of secondary metabolites. The substrate type or the source of carbon and nitrogen plays an important role in the production of *Monascus* pigments (MPs) and citrinin. For example, Lin and Demain^14^ showed that using monosodium glutamate, maltose, and an initial pH of 5.5 maximized the production of red MPs. In contrast, Jiang et al.^17^ found that ammonium salts enhanced yellow and orange MPs, outperforming nitrate and organic nitrogen sources. Furthermore, exposure to stress conditions can induce the overproduction of secondary metabolites.^18,19^ Studies in which different strains of the fungus were cultivated under stress conditions show that under a certain level of stress, stimulation of pigments and inhibition of citrinin production occurred.^20−23^ Additionally, stress conditions caused changes in production ratios of individual MPs and a shift of absorption maxima indicating the different composition of extracts.^23,24^

In this paper, *Monascus purpureus* DBM 4360, producing both C5- and C7-side chain pigments and mycotoxin citrinin, was cultivated using different carbon and nitrogen sources and under osmotic stress conditions in the presence of high concentrations of salt and/or high glucose. To the best of our knowledge, the correlation between pigment and citrinin production has not been observed in this way so far, but a correlation between the production of two yellow pigment analogues, ankaflavin and monascin, has already been found in *Monascus purpureus*.^25^ The range of conditions tested is based on previous experience with the strain.^26,27^ We focused on whether the ratio of five- to seven-carbon side chain pigments could be changed by culture conditions and whether the production of pigments and citrinin can be independently affected by different cultivation conditions.

## Materials and Methods

### Microbial Strain

*Monascus purpureus* DBM 4360 was used in this study. The strain is deposited in the Culture Collection of the Department of Biochemistry and Microbiology (DBM), University of Chemistry and Technology Prague, and was maintained on Sabouraud agar slants (VWR Chemicals, USA) at 4 °C. An inoculum spore (conidia) suspension was prepared by washing the grown mycelia (after 5 days culture) from agar slants with physiological solution containing 0.01% of Tween 80 and by scraping the surface with a sterile inoculation loop. The prepared spore suspension was transferred to a sterile tube and inoculated in a culture medium (1% v/v).

### Microscopy

Morphology of the mycelium and the localization of MPs was determined by phase-contrast microscopy (Olympus BX51) with ×400 magnification. The microphotographs were captured directly with a camera (EOS 600D, Canon). The microscopic preparation was made by transferring a part of the mycelium from an agar plate culture by an inoculation loop to a droplet of distilled water on a microscope slide.

### Culture Conditions

#### Cultivation Using Different Carbon and Nitrogen Sources

*Monascus purpureus* was cultivated in 24-well plates (see Supplement Figure S1) on a rotary shaker (Infors, 100 rpm) at 30 °C for 14 days. Stock solutions were sterilized by autoclaving (Tuttnauer ELV 3170, 121 °C, 20 min), and trace element stock solutions were sterilized by filtration. Stock solutions were mixed in each well to reach the required concentration at the final volume of 2 mL (g/L): potassium chloride (Lach-Ner, Czechia) 0.5, monopotassium phosphate (Penta, Czechia) 4, zinc sulfate heptahydrate (Penta, Czechia) 0.01, magnesium sulfate heptahydrate (Carl Roth, Germany) 0.5, iron(II) sulfate heptahydrate (Lach-Ner, Czechia) 0.01, and different combinations of carbon and nitrogen sources (Table 1). The initial pH was 5.5. Each carbon source was combined with each nitrogen source, and the concentrations were calculated to reach the same molar quantity of carbon/nitrogen. All chemicals used were practical grade (p.a.).

**Table 1:** List of Carbon and Nitrogen Sources

carbon source	g/L	nitrogen source	g/L
glucose (Lach-Ner, Czechia)	50.0	casamino acids (Gibco, USA)	8.55
rice starch (Sigma-Aldrich, USA)	50.0	monosodium glutamate (Fischer scientific)	14.10
fructose (Lach-Ner, Czechia)	50.0	ammonium sulfate (Penta, Czechia)	5.00
maltose (Fluka, Germany)	47.5	peptone (Carl Roth, Germany)	8.55
sucrose (Lach-Ner, Czechia)	47.5	sodium nitrate (Penta, Czechia)	6.41
glycerol (Lach-Ner, Czechia)	51.2	yeast extract (Merck, Germany)	8.54
lactose (Sigma-Aldrich, USA)	47.5	tryptone (Sigma-Aldrich, USA)	8.54
arabinose (Lachema, Czechia)	50.0	ammonium chloride (Lachema, Czechia)	4.27
xylose (Lachema, Czechia)	50.0		

After cultivation, the grown mycelium was separated from the culture broth by filtration using folded filter papers. Separated mycelia were used for extraction of intracellular secondary metabolites and determination of their production.

#### Cultivation under Stress Conditions

Cultivation was carried out in 250 mL Erlenmeyer flasks containing 100 mL of culture medium (see Supplement Figure S1) for 14 days at 30 °C on a rotary shaker (Infors, 100 rpm). Standard culture medium contains (g/L) glucose (Lach-Ner, Czechia) 50, ammonium sulfate (Penta, Czechia) 5, potassium chloride (Lach-Ner, Czechia) 0.5, monopotassium phosphate (Penta, Czechia) 4, zinc sulfate heptahydrate (Penta, Czechia) 0.01, magnesium sulfate heptahydrate (Carl Roth, Germany) 0.5, and iron(II) sulfate heptahydrate (Lach-Ner, Czechia) 0.01. Culture media were prepared with distilled water and sterilized by autoclaving (Tuttnauer ELV 3170, 121 °C, 20 min). The pH was adjusted to 5.5 with sodium hydroxide and hydrochloric acid (Penta, Czechia) solutions before sterilization. Different glucose and/or salt concentrations (see Table 2) were added to the standard culture medium to induce osmotic stress. All chemicals used were practical grade (p.a.).

**Table 2:** Concentrations of Glucose and Sodium Chloride (Lach-Ner, Czechia) in Stress Cultivation Media^a^

	stress condition													
	1	2	3	4	5	6	7	8	9	10	11	12	13	14
glucose [g/L]	50	50	50	50	50	50	50	50	100	100	100	100	100	150
sodium chloride [g/L]	5	20	35	50	70	100	125	150	0	35	50	70	100	0

a Standard culture medium (control) contained 50 g/L glucose and no sodium chloride.

After cultivation, grown mycelium was separated from the culture broth by filtration. Separated wet mycelium was weighed, and part of the mycelium was used for extraction of intracellular secondary metabolites and determination of their production. The pH of the culture broth was determined.

### Extraction of Secondary Metabolites

To determine the intracellular production of secondary metabolites, grown mycelium was extracted with 85% acidified (pH 4) ethanol, where the extraction ratio was 0.1 g of wet biomass: 1 mL of extraction solution. The extraction was carried out on a rotary shaker (Infors) at 30 °C for 40 min.

### Analysis of Secondary Metabolites

Total pigment production was evaluated based on the color of the extract by measuring the absorption spectra and finding the absorption maxima, as this is an established practice in the field and allows comparison with the work of other authors. The absorbance of ethanol extracts and culture broth was determined by a Tecan Reader at 300–600 nm. The samples with absorbance values exceeding 1 were diluted by extraction solution in the case of extracts or by distilled water in the case of culture broth.

In addition, UHPLC analysis of pigments and citrinin was performed following the protocol published in Husakova et al.^28^ Typical MPs (monascin, ankaflavin, rubropunctatin, monascorubrin, rubropunctamine, and monascorubramine) were identified based on their absorption spectra and retention times. Some unknown MPs were also detected; unknown MPs were identified as yellow, orange, or red MPs based on their absorption spectra and quantified as equivalents to their respective standards, i.e., monascin, rubropunctatin, and rubropunctamine. An example of a typical chromatogram is given in Figure S2.

### Determination of Glucose Utilization and Production of Primary Metabolites

In culture broths after cultivation in flasks, utilization of glucose and production of primary metabolites (ethanol, glycerol, and organic acids) were analyzed by HPLC (Agilent Technologies 1200 Infinity) under the following conditions: Watrex 250 × 8 mm Polymer IEX H+ 8 μm column; mobile phase, 5 mM H_2_SO_4_ in demineralized water; isocratic elution at a flow rate 1 mL/min at 60 °C; injection volume 20 μL; and analysis run for 16 min. For the determination of compounds, the refractometric index detector was used. Pure compounds were used as standards.

### Statistical Analysis

Two-way ANOVA analysis with Tukey’s multiple comparison test was performed for cultivation using different carbon and nitrogen source data. One-way ANOVA analysis with Dunnett’s multiple comparisons test was performed for stress cultivation data. Simple linear regression and Spearman correlation were performed to determine the relationships between the production of MP pairs and between the production of MPs and citrinin. Spearman correlation was chosen according to the results of a normality test. Statistical analysis was performed using Graphpad Prism software. Significance levels were set up as *p < 0.05, **p < 0.01, and ***p < 0.001.

## Results

### Impact of Different Carbon and Nitrogen Sources on MPs and Citrinin Production in Small Scale Cultivations

At first, small scale cultivations were carried out in 24-well plates with different combinations of nitrogen and carbon sources, in all cases maintaining their relative molar concentration ratios, and the effects on MPs and citrinin production were assessed. In the field, there is no gold standard on how to evaluate secondary metabolite production by the fungus *Monascus*. We decided to evaluate separately the intracellular and extracellular production of secondary metabolites and used mycelial extracts (extraction was carried out under conditions where the “artificial, nonbiosynthetic” production of red pigments was limited by the pH of the extraction reagent) for the former and postculture medium for the latter. We evaluated separately the total color by spectrophotometry and the individual substances by UHPLC. However, since the absorbance of the culture medium was several times less than that of the mycelial extracts and no peaks corresponding to standard MPs or citrinin were detected by UHPLC analysis, extracellular production was neglected, and the following results reflect only intracellular production.

**Figure 1:**
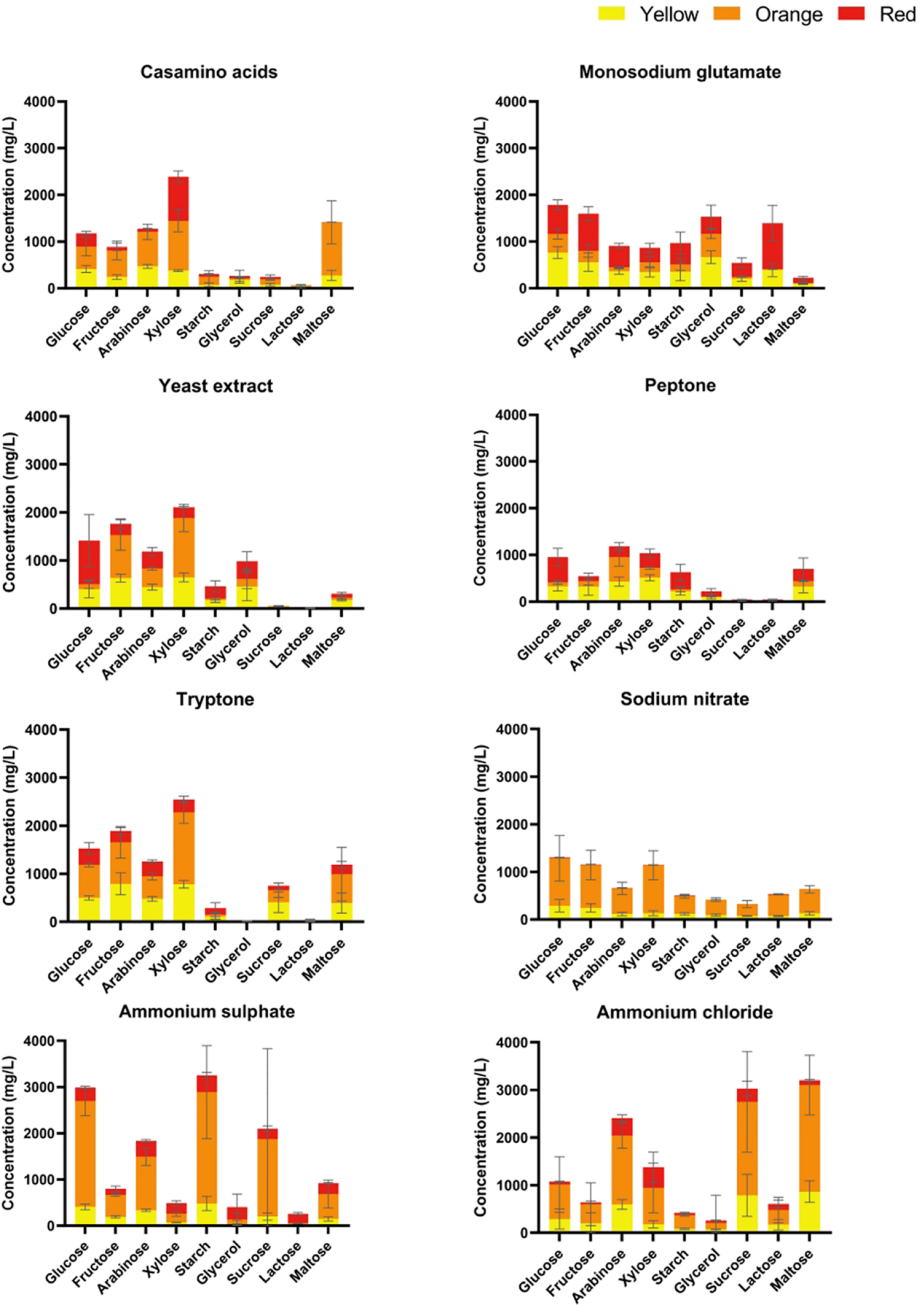
Concentrations of all MPs produced in 24 well plates with different carbon and nitrogen sources determined by UHPLC analysis grouped according to the nitrogen source. All cultivations were performed in triplicate, and the data are presented as the mean of three values with standard deviations. Statistical differences between MP production under each cultivation condition are stated in Supporting Information Table S1.

**Figure 2:**
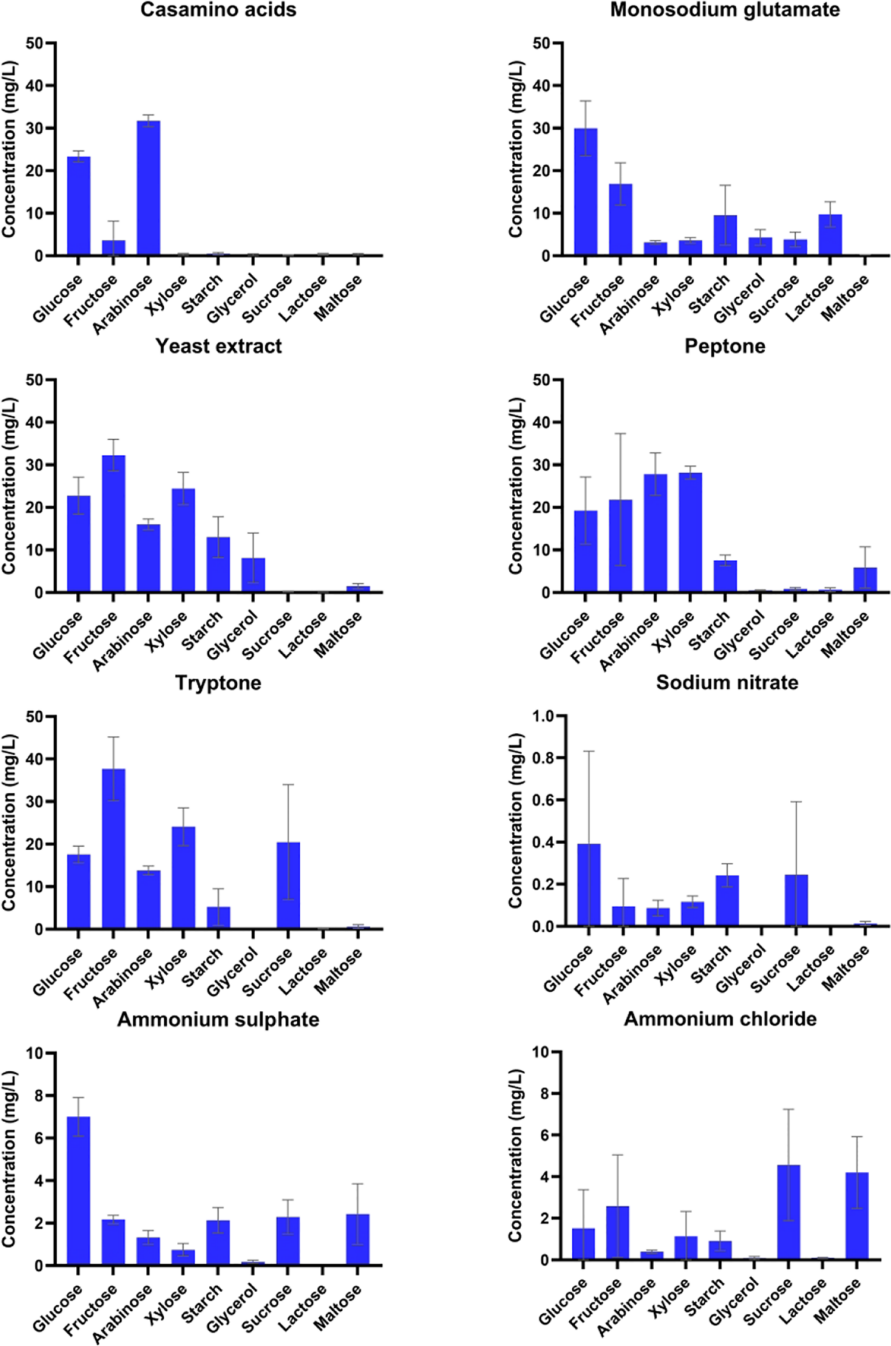
Concentrations of citrinin determined in the extracts of the grown mycelia. All cultivations were performed in triplicate, and data are presented as the mean of three values with standard deviations. Statistical differences between citrinin production under each cultivation condition are stated in Supporting Table S1. Graphs have different scales of *Y* axes.

The production of individual MPs under cultivation with different carbon and nitrogen sources is shown in Figure 1. Typical MPs (yellow–ankaflavin, monascin; orange–rubropunctatin, monascorubrin; and red–rubropunctamine and monascorubramine) were detected together with up to three unidentified yellow MPs, four unidentified orange MPs, and four red MP derivatives in all extracts (a typical chromatogram is shown in Supplement Figure S2). The MP production is presented as a sum of all detected MPs having yellow, orange, or red color. Citrinin production under the same conditions is shown in Figure 2. Higher MPs and citrinin productions were detected in cultivations with monosaccharides, but citrinin production was 100–1000 times lower compared to pigments, depending on the composition of the culture medium. Higher amounts of orange MPs were detected using glucose, starch, and sucrose in combination with (NH_4_)_2_SO_4_, and sucrose and maltose in combination with NH_4_Cl, which corresponds well with the final pH reached in these cultivations (see Supplement Figure S3). At pH below 4, the production of red pigments from orange ones was inhibited. The red pigments, in contrast to yellow and orange ones, are considered not to be formed biosynthetically but by the reaction between orange pigments and amino group-containing compounds.

Further, the amount of wet biomass produced and the absorption spectra of mycelium extracts reflecting their coloration were evaluated and are shown in Figures S4 and S5. Absorption spectra comply with the UHPLC findings with maxima distributed mainly in areas around 410, 470, and 500 nm. These wavelengths correspond to the combined effect of the overlap in the absorption of yellow, orange, and red MPs (see e.g., Husakova et al.^28^) and are shifted according to the occurrence and predominance of a particular group of pigments. The highest absorbance occurred with extracts of arabinose in combination with NH_4_Cl (maxima: 54.34 at 418 nm and 48.00 at 472 nm) and xylose in combination with yeast extract (maxima: 54.21 at 416 nm and 47.72 at 486 nm).

**Figure 3:**
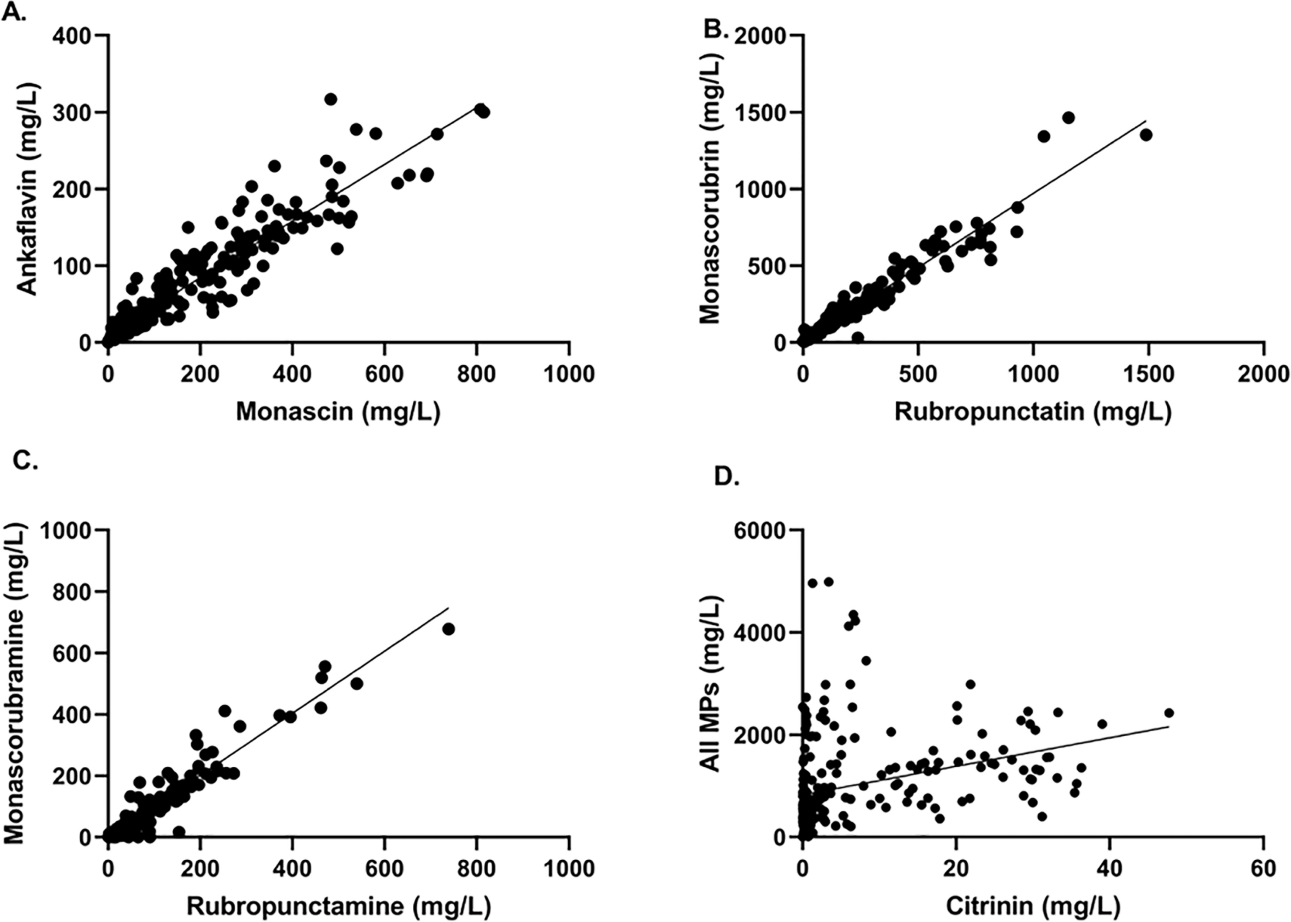
Graphical relationships of individual MPs and citrinin produced in cultivations with different carbon and nitrogen sources: (A) yellow MPs analogues monascin (C5) to ankaflavin (C7), (B) orange MPs analogues rubropunctatin (C5) to monascorubrin (C7), (C) red MPs analogues rubropunctamine (C5) to monascorubramine (C7), and (D) citrinin to all MPs production. Each spot represents one parallel culture experiment spot, and spots were fitted with a linear regression curve. Graphs have different scales of the *X* and *Y* axes.

To determine whether there was any relationship between the production of individual pigments in five- and seven-carbon side chain pairs, between groups of pigments (yellow, orange, and red), and between pigments and citrinin, we plotted the amounts of these compounds against each other. Linear dependence was observed between the production of five- and seven-carbon side chain analogues not only in the case of yellow and orange MP pairs but also for the red ones (see Figure 3). The equations and R squared values of the linear regression curves and the Spearman correlation coefficients are stated in Table 3, also for other comparisons, such as yellow to all MPs or orange to all MPs. While a highly probable link was found between yellow and orange MPs mutually and to all MPs, production of red MPs seemed to be independent of yellow or orange MP production. To analyze the dependence of citrinin and MP production, the sum of all MP concentrations was correlated with citrinin (Figure 3D). Other plots of MPs to citrinin are presented in Supplementary Figure S6.

**Table 3:** Equation and *R* Squared Value of the Linear Regression Curves and the Spearman Correlation Coefficients of MPs and Citrinin Produced in Small Scale Cultivation with Different Carbon and Nitrogen Sources

relationship	simple linear regression		correlation	
	*R*^2^	equation	spearman correlation coefficient r	**correlation definition by Chan**(29)
C5:C7 analogues	0.9302	*y* = 0.7345*x* + 13.72	0.9684	very strong
monascin:ankaflavin	0.8608	*y* = 0.3694*x* + 10.47	0.9376	very strong
rubropunctatin:monascorubrin	0.9442	*y* = 0.9653*x* + 6.521	0.9817	very strong
rubropunctamine:monascorubramine	0.9218	*y* = 1.012*x* – 0.9151	0.9354	very strong
yellow MPs:all MPs	0.6602	*y* = 3.042*x* + 136.9	0.9005	very strong
orange MPs:all MPs	0.813	*y* = 1.238*x* + 399.7	0.8479	very strong
red MPs:all MPs	0.2078	*y* = 1.645*x* + 655.8	0.6203	moderately strong
yellow MPs:orange MPs	0.3269	*y* = 1.559*x* + 51.30	0.6706	moderately strong
yellow MPs:red MPs	0.00558	*y* = 0.02842*x* + 212.9	0.2073	poor
orange MPs:red MPs	0.2168	*y* = 0.4829*x* + 85.57	0.6447	moderately strong
citrinin:all MPs	0.1014	*y* = 27.89*x* + 825.9	0.5572	fair
citrinin:yellow + orange MPs	0.06129	*y* = 19.68*x* + 658.6	0.4595	fair
citrinin:yellow MPs	0.3867	*y* = 14.55*x* + 187.2	0.7494	moderately strong
citrinin:orange MPs	0.00648	*y* = 5.134*x* + 471.4	0.2574	poor
citrinin:red MPs	0.1144	*y* = 8.209*x* + 167.4	0.6324	moderately strong

The close correlation was confirmed by calculating the Spearman correlation coefficient (Table 3) between the production of pigments in pairs with different side chain lengths (Figure 3); a similar correlation as for orange pigments was also found for the red pigments monascorubramine and rubropunctamine, which are assumed to have a nonbiosynthetic origin. However, in the case of pigments and citrinin, it is not possible to conclusively say from the results whether there is a relationship between their biosynthesis. Their apparently independent production (Figure 3 and Supplement Figure S6) was not clearly confirmed by the calculation of the Spearman coefficient (Table 3).

### Large-Scale Cultivation under Stress Conditions and Impact on Primary and Secondary Metabolism

To further analyze the interrelationships between pigment and citrinin production, we performed a series of larger-scale experiments in Erlenmeyer flasks under stress conditions that should lead to the induction of more fundamental changes in secondary metabolite production and could allow us to analyze them in more detail.

**Figure 4:**
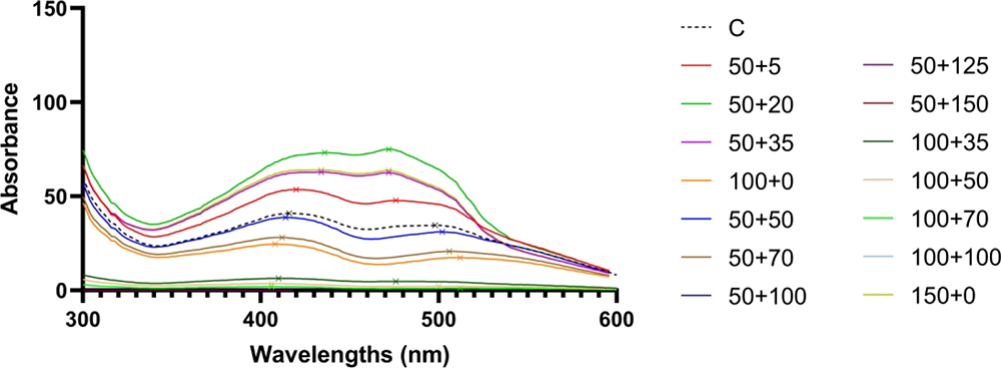
Absorption spectra (300–600 nm) of mycelial extracts. (×) represents the values of the absorption maxima. All cultivations were performed in triplicate; data are presented as a mean of three values. Labeling of samples: C – standard cultivation conditions (control); stress cultivation conditions, as written in Table 2; the first labeling number corresponds to initial glucose concentrations (50; 100; 150 g/L) + second labeling number corresponds to sodium chloride concentrations (5–100 g/L).

The total color of the samples corresponding to the content of all dissolved substances was determined at first spectrophotometrically (see Figure 4). The absorption spectra indicated the presence of all MP types and under some stress conditions (50 + 5, 50 + 20, 50 + 35, 100 + 35, 150 + 0) shifts of absorption maxima from 498 to 472 nm were observed.

**Figure 5:**
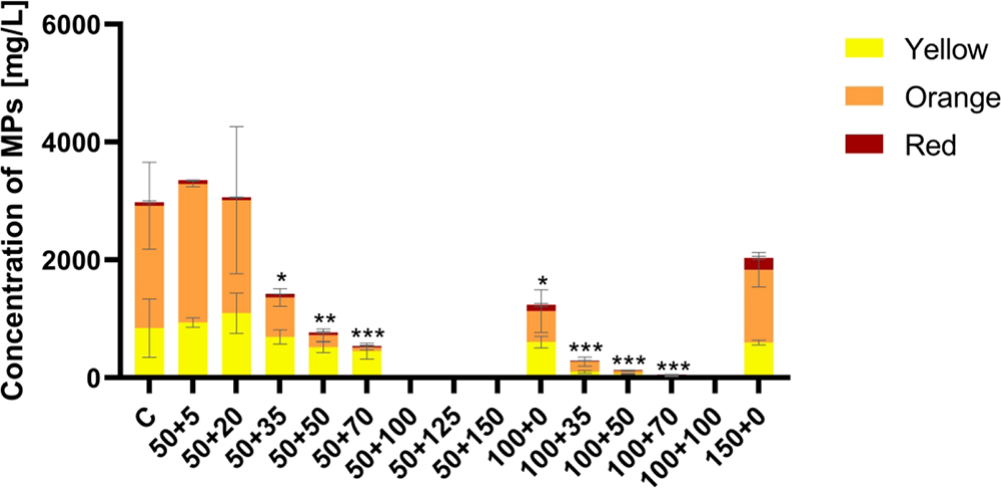
Production of MPs by a *Monascus purpureus* DBM 4360. All cultivations were performed in triplicate, data are presented as the mean of three values with standard deviation, statistical significance: *p < 0.05; **p < 0.01; ***p < 0.001. Labeling of samples: C – standard cultivation conditions; stress cultivation conditions, as written in Table 2; the first labeling number corresponds to initial glucose concentration (50; 100; 150 g/L) + second labeling number corresponds to sodium chloride concentration (5–100 g/L).

**Figure 6:**
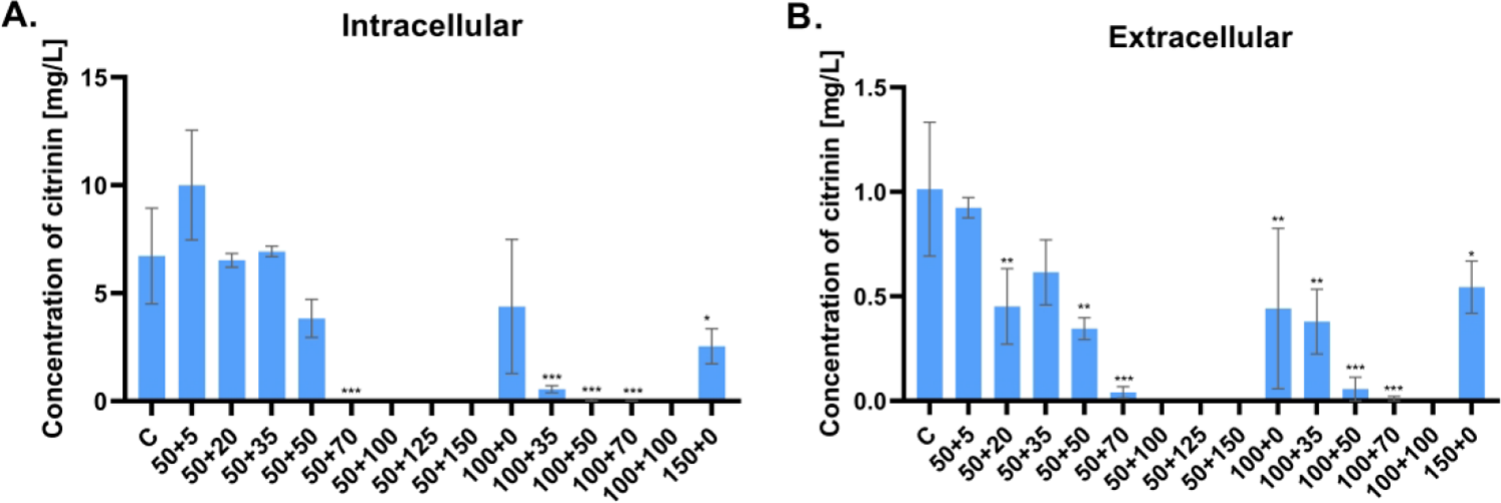
Concentrations of citrinin produced by a *Monascus purpureus* DBM 4360. (A) Intracellular concentrations determined in the extracts of grown mycelia; (B) extracellular concentrations determined in filtered culture broth. All cultivations were performed in triplicate, data are presented as the mean of three values with standard deviation, statistical significance: *p < 0.05; **p < 0.01; ***p < 0.001. Labeling of samples: C - standard cultivation conditions; stress cultivation conditions, as written in Table 2; the first labeling number corresponds to initial glucose concentration (50; 100; 150 g/L) + second labeling number corresponds to sodium chloride concentration (5–100 g/L). Graphs have different scales of *Y* axes.

Furthermore, UHPLC analysis was performed, and typical MPs (ankaflavin, monascin, rubropunctatin, monascorubrin, rubropunctamine, and monascorubramine), two unknown yellow MPs, and some unknown orange MPs (the number differed depending on stress conditions), together with citrinin, were quantified. In the extracts, all types of MPs were detected (Figure 5). Under most of the conditions, the major MPs were orange, accompanied by yellow MPs and a small number of red MPs. The production of mycotoxin citrinin was determined by UHPLC in culture broth (Figure 6B) and in extracts (Figure 6A), and its production did not correlate with increasing stress.

In all samples of culture broth, no peaks corresponding to the typical MPs were detected by UHPLC analysis, and the absorption maxima were in the range of 350–390 nm, although the broth color was orange–red (data not shown).

**Figure 7:**
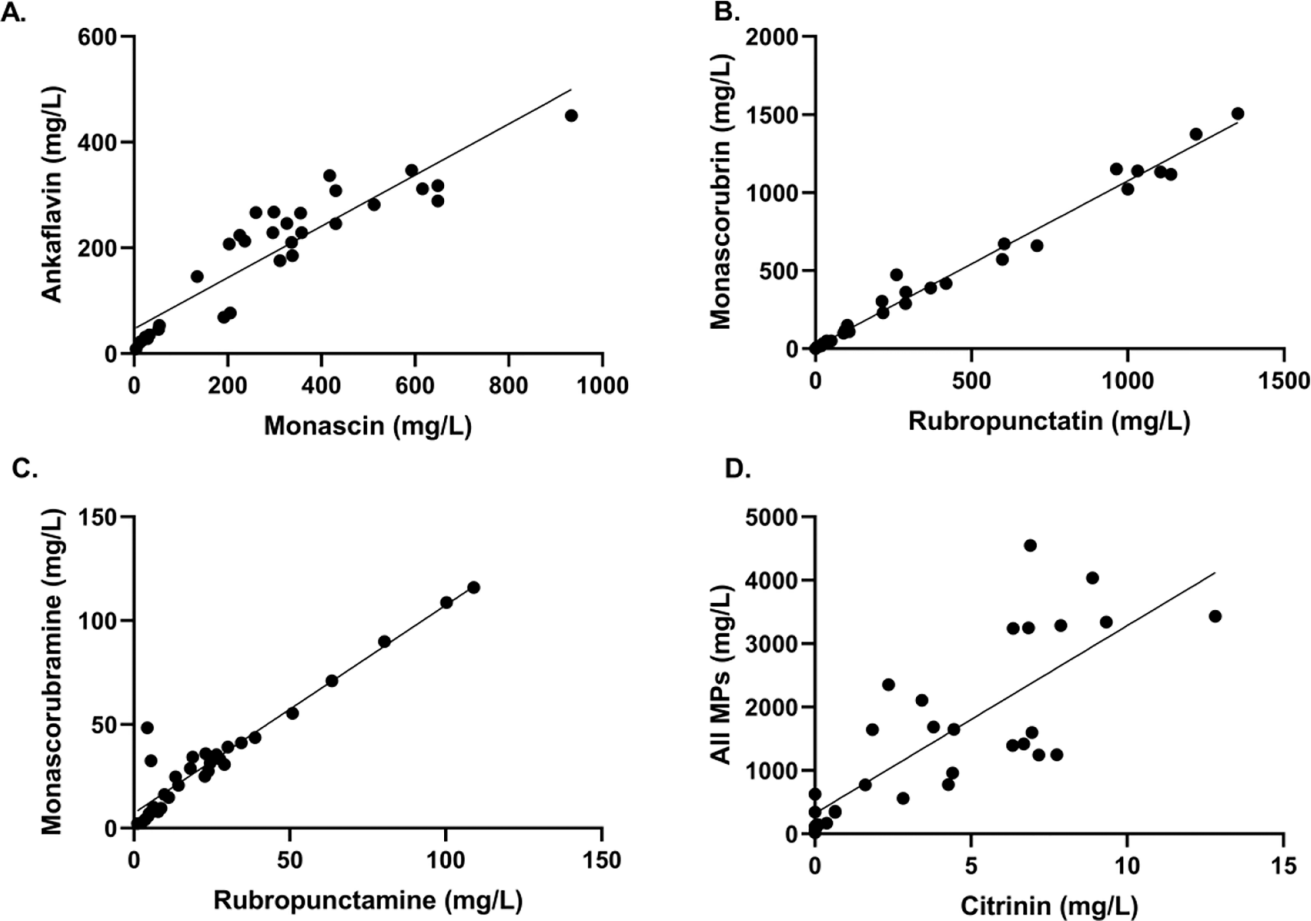
Graphical relationships of individual MPs and citrinin in extracts under stress conditions: (A) yellow MPs analogues monascin (C5) to ankaflavin (C7), (B) orange MPs analogues rubropunctatin (C5) to monascorubrin (C7), (C) red MPs analogues rubropunctamine (C5) to monascorubramine (C7), and (D) citrinin to all MPs production. Each spot represents parallel culture experiment spot; the spots were fitted with a linear regression curve. Graphs have different scales of *X* and *Y* axes.

The relationships between five- and seven-carbon side chain analogues, MPs, and citrinin production are shown in Figures 7 and S7. The linear dependence between five- and seven-carbon side chain analogues, between yellow and all MPs, and orange and all MPs production was confirmed. However, the relationships between MPs and intracellular production of citrinin also showed moderately to very strong correlations (see Table 4).

**Table 4:** Equations and *R* Squared Values of the Linear Regression Curves and the Spearman Correlation Coefficients of MPs and Citrinin Produced in Large Scale Cultivation under Stress Conditions

relationship	simple linear regression		correlation	
	*R*^2^	equation	spearman correlation coefficient r	correlation definition by Chan^29^
C5:C7 analogues	0.971	*y* = 0.8376*x* + 41.83	0.983	very strong
monascin:ankaflavin	0.8435	*y* = 0.4849*x* + 46.73	0.9288	very strong
rubropunctatin:monascorubrin	0.9854	*y* = 1.063*x* + 12.07	0.988	very strong
rubropunctamine:monascorubramine	0.9113	*y* = 1.001*x* + 7.227	0.8476	very strong
yellow MPs:all MPs	0.8433	*y* = 3.141*x* – 255.9	0.9231	very strong
orange MPs:all MPs	0.9727	*y* = 1.360*x* + 294.5	0.9756	very strong
red MPs:all MPs	0.1464	*y* = 9.048*x* + 920.4	0.7453	moderately strong
yellow MPs:orange MPs	0.7119	*y* = 2.093*x* – 287.7	0.87	very strong
yellow MPs:red MPs	0.1131	*y* = 0.01960*x* + 41.05	0.7209	moderately strong
orange MPs:red MPs	0.1088	*y* = 0.04770*x* + 31.81	0.6193	moderately strong
citrinin:all MPs	0.6479	*y* = 296.1*x* + 322.5	0.8338	very strong
citrinin:yellow + orange MPs	0.6522	*y* = 292.5*x* + 278.5	0.8389	very strong
citrinin:yellow MPs	0.6474	*y* = 86.52*x* + 213.3	0.8067	very strong
citrinin:orange MPs	0.5959	*y* = 205.9*x* + 65.18	0.8586	very strong
citrinin:red MPs	0.05388	*y* = 3.610*x* + 43.94	0.6332	moderately strong

**Figure 8:**
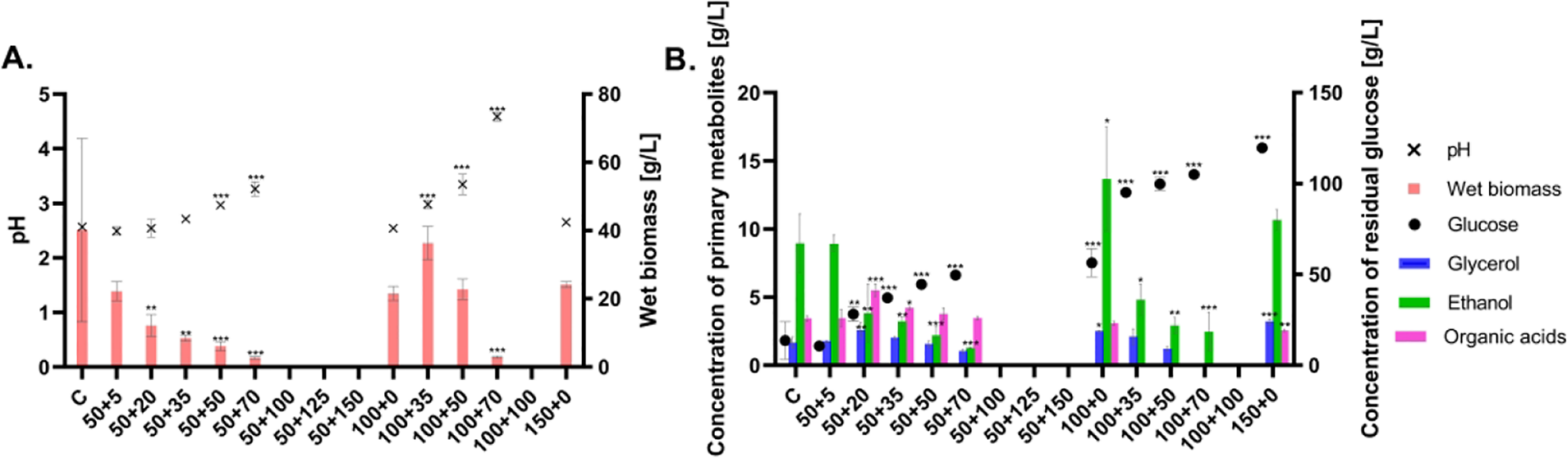
Biomass and final pH (A) and production of primary metabolites and consumption of glucose (B). All cultivations were performed in triplicate, data are presented as the mean of three values with standard deviation, statistical significance: *p < 0.05; **p < 0.01; ***p < 0.001. Labeling of samples (on *x* axes): C – standard cultivation conditions (control), stress cultivation conditions, as written in Table 2; the first labeling number corresponds to initial glucose concentration (50; 100; 150 g/L) + second labeling number corresponds to sodium chloride concentration (5–100 g/L).

As can be seen in Figure 8, increasing osmotic pressure had an impact on growth by decreasing the amount of biomass produced, and the higher final pH values of culture broth and the inhibition of growth were mainly due to the high salt concentration, not glucose. The growth parameters were evaluated as wet biomass; the moisture of biomass was between 69 and 84% (data not shown). The production of ethanol was observed even under aerobic conditions, the highest concentrations being 10–14 g/L produced in a surplus of glucose (100–150 g/L). Glycerol as an osmoprotective agent was produced at concentrations around 3 g/L. Consumption of glucose and biomass production was concerted; both these parameters followed the trend of increasing osmotic stress. The production of organic acids (malic, succinic, and fumaric) was detected under almost all conditions, with the highest concentration being obtained under stress conditions with 50 g/L of glucose and 20 g/L of sodium chloride (5.5 g/L), and with 50 g/L of glucose and 35 g/L of sodium chloride (4.2 g/L).

## Discussion

This study provides insights into the relationships between the production of individual MPs and between MPs and citrinin under different conditions. Correlations between the formation of five- and seven- carbon side chain MP analogues found under all culture conditions testify to the relatively strict regulation of the production of individual analogues, which is probably governed by MpFasA2 and MpFasB2.^30^ In *Monascus ruber*, the enzymes are known as MrPigJ and MrPig K^31^. The mutual relationship between yellow pigments ankaflavin and monascin has already been described by Lin et al.,^25^ but our research extended the conclusions to also be valid for orange and red pigments. In the case of the pair of two basic red pigments (monascorubramine and rubropunctamine), this is surprising because they are not supposed to be biosynthesized. Moreover, orange pigments can react with a wide range of substances containing an available amino group (e.g., amino acids)^32^ under a suitable pH, and in addition to the two basic red MPs (for which the correlation was evaluated), a heterogeneous group of substances with similar spectral characteristics can be formed. The correlation found between the two basic red pigments indicates that they may have formed by regulated biosynthesis as already suggested by Chai et al.^33^ Nevertheless, this correlation can be explained by both biosynthetic and chemical hypotheses since the formation of these pigments corresponds to the availability of reactants (orange pigments) in the same proportion. This leaves open the possibility of both mechanisms. Regarding the other red pigment derivatives, their formation might be a matter of chance, i.e., the simultaneous presence of reactants at a suitable pH.

**Figure 9:**
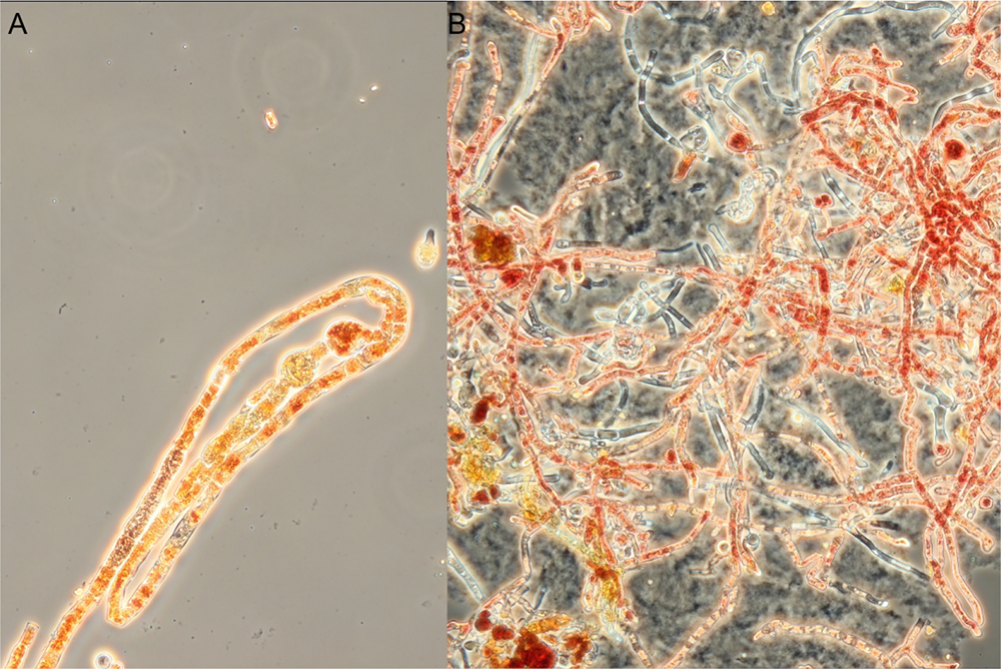
Distribution of orange/red pigments in hyphae (A) and mycelia (B) of *Monascus purpureus* DBM 4360 revealing the localization of orange and red MPs as well as hyphae without pigments, magnification 400×.

Recently, it was hypothesized^31^ that the formation of orange MPs from their precursor, catalyzed by MrPigF (oxidoreductase), is compartmentalized into a cell wall. The compartmentalization of formation of orange MPs actually seems to be a necessity because if they were formed in the cytosol, they would be rapidly converted to red MP derivatives, and the reaction might interfere with the whole range of cell functions. However, our observations of orange and red pigments in the *M. purpureus* DBM 4360 hyphae or mycelia under the microscope are not consistent with potential cell wall localization. In addition to the obvious heterogeneous presence of pigments in various hyphae (Figure 9), Lu et al.^34^ described the accumulation of orange and red *Monascus anka* pigments in the form of extracellular crystals. All of these inconsistent data leave unresolved gaps in the understanding of biosynthesis.

A correlation was also found between the total production of yellow and orange MPs, which indicates tight regulation of their biosynthesis. In *Monascus ruber*, the same intermediate is assumed for both groups, and the enzymes involved in their biosynthesis are MrPigH (enoyl reductase) and MrPigF (FAD-dependent oxidoreductase),^10^ respectively. Surprisingly, it was not possible to find culture conditions causing a significant change among production of MP groups, i.e., which would lead to overproduction of one group (yellow, orange, red) of pigments at the expense of the others. This is in contrast to the results published by other authors.^35−37^ Although the culture medium after culture was often strongly colored and the absorption spectra were comparable to those of the extracts, no pigment from the typical six could be found by UHPLC. Therefore, it seems obvious that water-soluble pigments share a similar chromogenic core but are different from classical MPs^24^ and require different analytical conditions for their determination.^38^

Regarding the use of various combinations of carbon and nitrogen sources, monosaccharides proved to be the most effective carbon source for total MP (yellow + orange + red) production, followed by starch in combination with (NH_4_)_2_SO_4_. Ammonium salts ((NH_4_)_2_SO_4_ and NH_4_Cl) were found to be the best nitrogen sources for overall MP production, confirming the results obtained by Liu et al.^39^ In contrast, Jiang et al.^17^ and Chen and Johns^40^ found organic nitrogen sources (peptone, yeast extract, or beef extract) more convenient for MP production than inorganic salts including ammonium salts. Overall, all MP groups were present under most cultivation conditions, except for cultivation with NaNO_3_, where only yellow and orange MPs were detected. The reaction of orange MPs and the formation of red ones did not occur even if the pH was suitable for the reaction (in the range of 4–6) because the broth did not contain any convenient compounds with amino groups for the reaction.

In different studies,^20−23^ stimulation of MP production by high-salt (up to 120 g/L of sodium chloride) and/or by high-glucose (up to 200 g/L of glucose) stress was found. In addition, changes in MP production ratios were manifested as shifting of absorption maxima in pigment extracts under stress conditions.^23,24^ Although stimulation of pigment production by stress conditions was not confirmed in our study, a shift in the absorption maxima from red to orange MPs was observed in some extracts. With increasing concentrations of sodium chloride and glucose, a decreasing trend in the production of pigments was observed. Regarding mycotoxin production under stress conditions, different authors reached different results, i.e., both overproduction^18^ or inhibition (in the case of citrinin production if addition of sodium chloride ((up to 0.4 M sodium chloride, i.e., about 23 g/L)) to *M. purpureus* cultivation was used).^22^ In our case, in different *M. purpureus* strains, suppression of total (intracellular + extracellular) citrinin production was observed by addition of 70 g/L sodium chloride; by addition of 100 g/L glucose in combination with 35, 50, and 70 g/L sodium chloride; and by addition of 150 g/L glucose, while other stress conditions resulted in a comparable amount of citrinin to control.

After the first series of experiments with different carbon and nitrogen sources in microwell plates, relationships between the production of citrinin and the production of yellow or orange MPs were not found. The mycotoxin citrinin remains a persistent problem that hinders research and the use of *Monascus* natural pigments in the food industry.^41^ If the formation of MPs and citrinin was not connected, there would be great hope for the future that pigment-hyperproducing strains of *M. purpureus* could be found/improved to not produce citrinin. However, these findings have not been confirmed in cultivations under stress conditions, where moderate to strong correlations were determined. One of the differences between the first and second series of experiments was differences in the morphology of the fungal mycelia (growth on the liquid surface in the first case and submersed growth in the form of pellets in the second case) and thus probably also in oxygen availability. The effect of oxygen availability on citrinin production by *M. ruber* has already been described^42^ as well as the influence of mycelium pellet size on citrinin production in *M. purpureus*.^43^

It is speculated that the key intermediate of both metabolic pathways is a tetraketide synthesized by polyketide synthases MrPigA^10^ or PksCT^12^ in the case of MPs and citrinin, respectively, or that some genes (i.e., *MptirA* and *ctnF*) are shared by both pathways.^13^ The question arises whether the two biosynthetic pathways for MPs and citrinin can cooperate by using an intermediate produced by the other pathway if their tetraketide intermediate is similar/same. Some findings, such as persistent citrinin production even after *pksCT* gene knockout^33^ or low expression levels of this gene during citrinin production,^11^ support this unconfirmed possibility. On the other hand, the order of magnitude differences in the produced amounts of the two secondary metabolites groups (with citrinin being produced at levels 100 times lower than MPs) call into question the possibility of common regulation or mutual cooperation of biosynthetic pathways.

A series of *Monascus purpureus* DBM 4360 cultivations with different carbon and nitrogen sources and also under osmotic stress were performed to investigate the relationship between citrinin and MP production and between production of MP analogues that differ in side chain length. It was found that there was a correlation (Spearman correlation coefficient *r* above 0.93) between the production of five- and seven-carbon side chain MP analogues of all color variants, including the red–monascorubramine and rubropunctamine, under all culture conditions tested. The results thus show that the incorporation of fatty acids of five or seven carbons into tetraketide is subject to strong regulation, the nature of which is an interesting target for future research. On the contrary, the relationship between MPs and citrinin production showed a less robust correlation, with a stronger correlation found for experiments under stress conditions, while a moderate to poor correlation was found in the broader experiment with different substrates. Thus, the mutual independence of MPs and citrinin biosynthesis has not been demonstrated nor can it be clearly stated that there is a relationship between them. However, our results indicate that citrinin production might be decoupled from MP production under certain conditions, offering the promise of producing MPs without citrinin contamination. Further research is needed to explore the potential to optimize these conditions, opening the way for the safe use of these pigments in the food industry.

## Supporting Information Available

Additional information (photographs from both types of cultivations, typical chromatograms of secondary metabolites including identification of individual peaks) and other evaluated data used in the paper, namely, final pH during cultivation on individual carbon sources, concentration of wet biomass after cultivation, absorption spectra of mycelial extracts, and graphical correlations between citrinin production and different groups of pigments (PDF)
Tukey’s multiple comparisons test (XLSX)
