# Supplementary material for: Coordinated Synthesis of Pigments Differing in Side Chain Length in Monascus purpureus and Investigation of Pigments and Citrinin Relation
Source: J Agric Food Chem. 2025 Jan 10;73(3):2033–43. doi: 10.1021/acs.jafc.4c09653 (PMC11760163; doi:10.1021/acs.jafc.4c09653)
Supplement: Supplementary file 1 — jf4c09653_si_001.pdf [file jf4c09653_si_001.pdf]

Supporting Information

Coordinated synthesis of pigments differing in side chain length in *Monascus purpureus* and investigation of pigments and citrinin relation

Marketa Husakova[1] https://orcid.org/0000-0001-6120-307X, Barbora Branska[1] https://orcid.org/0000-0001-6536-7063, Petra Patakova[1,*] https://orcid.org/0000-0002-9410-4454

[1]*Department of Biotechnology, University of Chemistry and Technology Prague, Technicka 5, Prague, CZ166 28, Czechia*

[*]Corresponding author: petra.patakova@vscht.cz

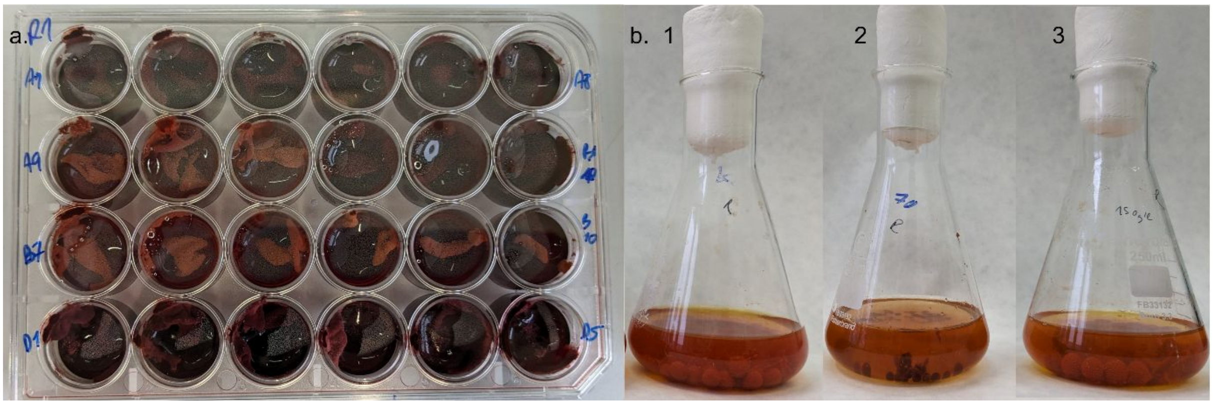

Figure S1. Representative photos of grown mycelia in 24 well microculturing plates (a) and in flasks (b); 1. Control (standard cultivation conditions), **2.** Stress cultivation conditions 50 g/L glucose and 70 g/L NaCl, **3.** Stress cultivation conditions 150 g/L glucose and 0 g/L NaCl. All cultivations were performed at 30°C, for 14 days, on rotary shaker.

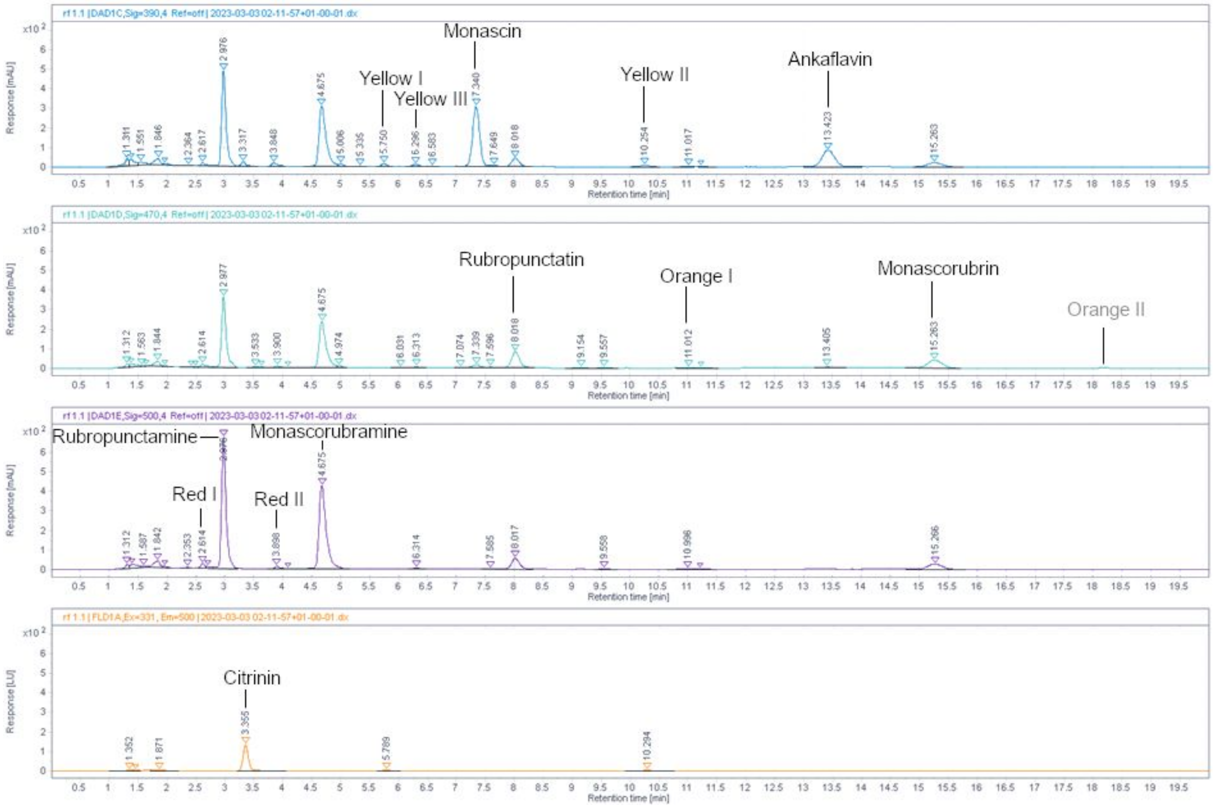

Figure S2. Typical chromatograms obtained by UHPLC analysis. DAD detector set up at 390 nm; 470 nm and 500 nm for the MPs analysis, and FLD detector set up at 331 nm for excitation and 500 nm for emission in case of citrinin determination.

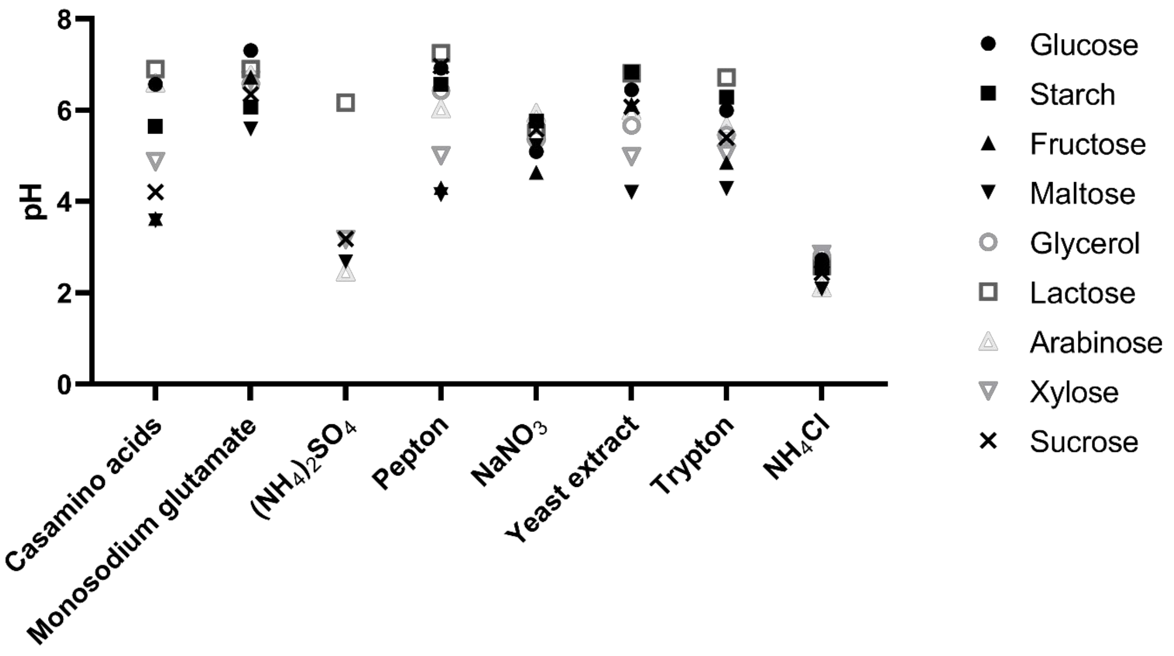

Figure S3. pH values of culture broth determined at cultivations with different sources of carbon and nitrogen sources.

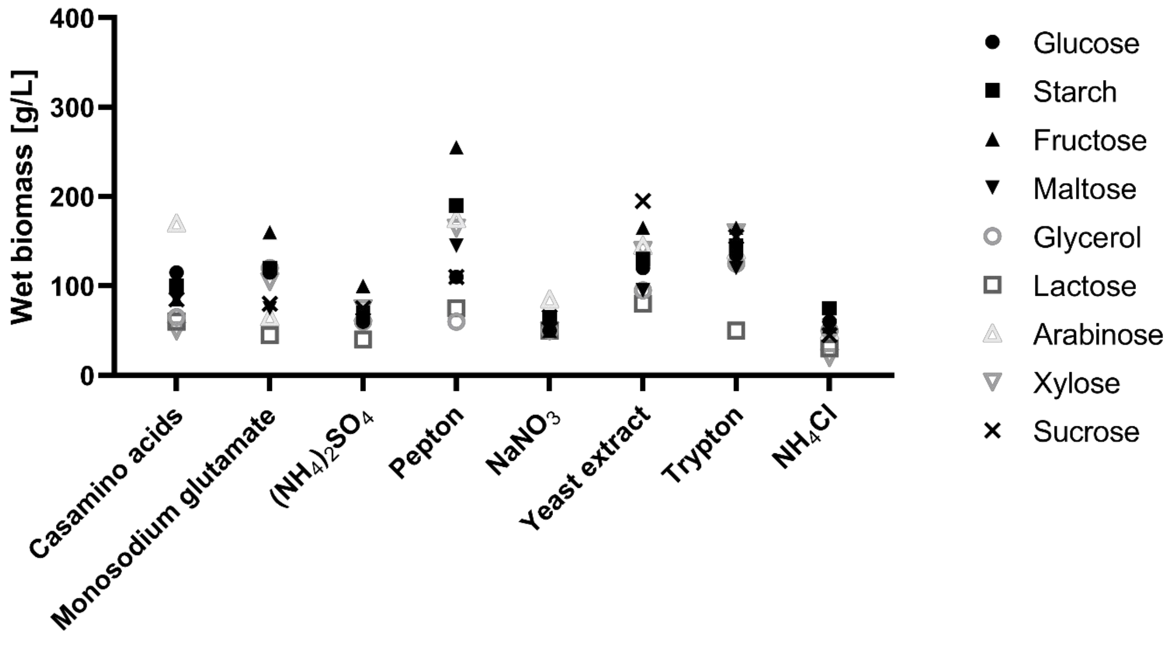

Figure S4. Wet biomass obtained from cultivations with different carbon and nitrogen sources.

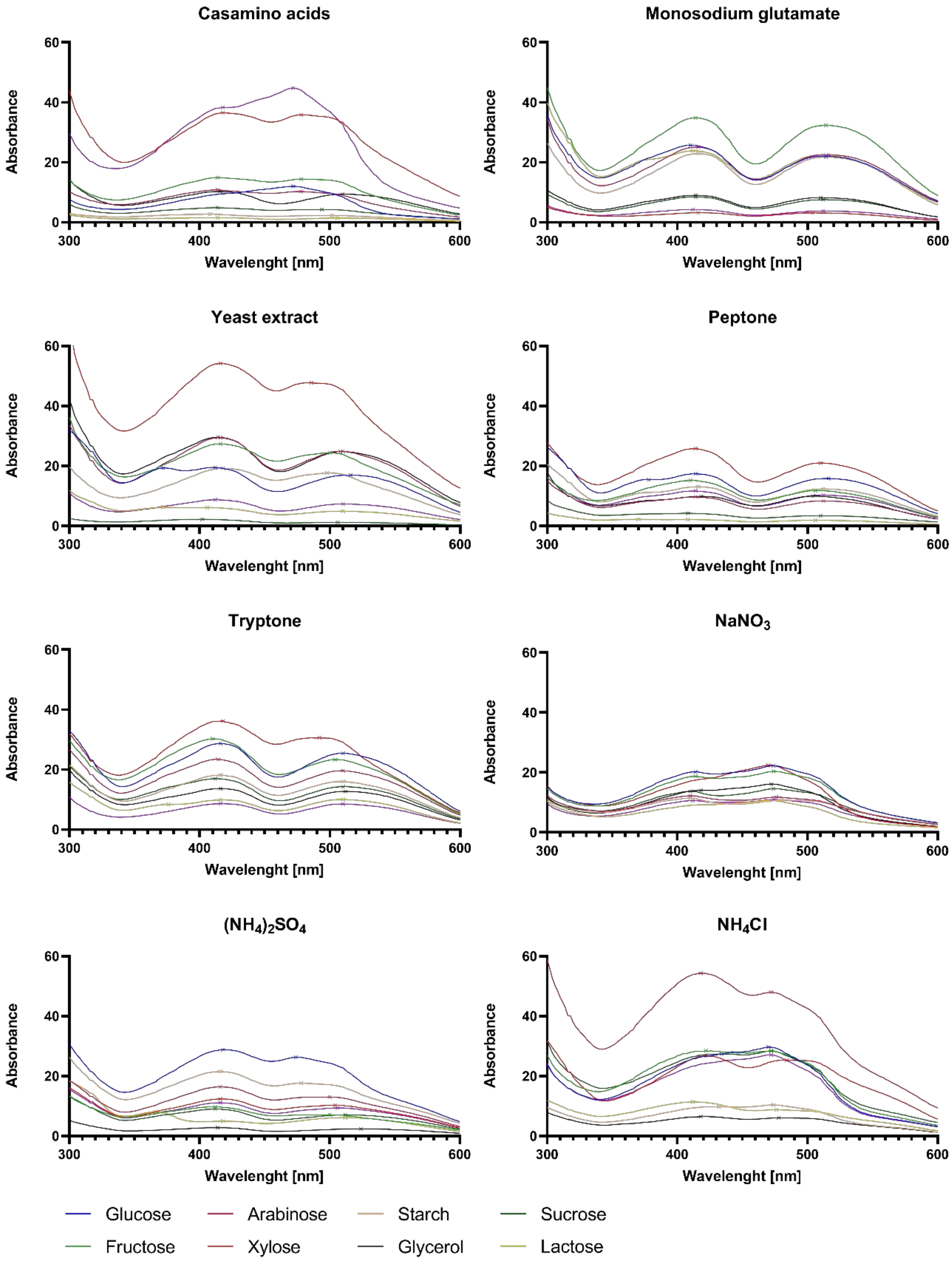

Figure S5. Absorption spectra (300-600 nm) of mycelia extracts. (×) represent the values of the absorption maxima. All cultivations were performed in triplicate, data are presented as a mean of three values.

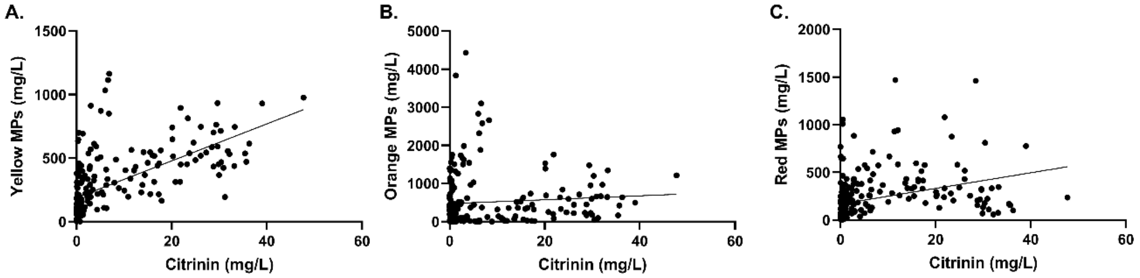

Figure S6. Graphical relationships between citrinin and MPs production from cultivations with different carbon and nitrogen sources. **A.** Citrinin to yellow MPs production; **B.** Citrinin to orange MPs production; **C.** Citrinin to red MPs production.

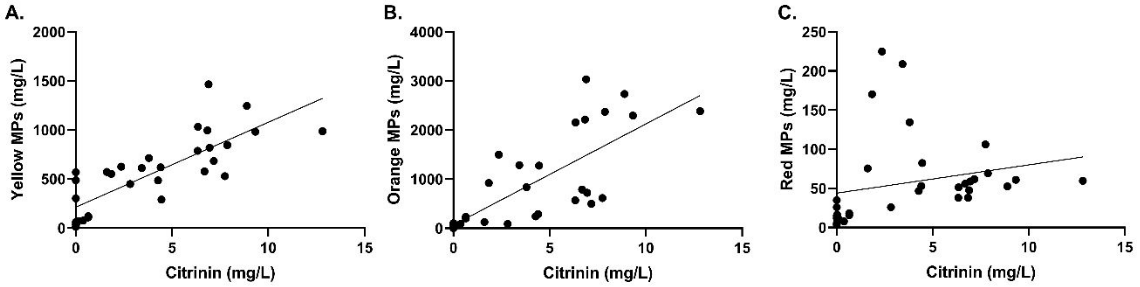

Figure S7. Graphical relationships between citrinin and MPs production from cultivations under stress conditions. **A.** Citrinin to yellow MPs production; **B.** Citrinin to orange MPs production; **C.** Citrinin to red MPs production.
